# Zoonotic *Ancylostoma ceylanicum* Hookworm Infections, Ecuador

**DOI:** 10.3201/eid2809.220248

**Published:** 2022-09

**Authors:** William J. Sears, Jorge Cardenas, Joseph Kubofcik, Thomas B. Nutman, Philip J. Cooper

**Affiliations:** National Institutes of Health, Bethesda, Maryland, USA (W.J. Sears, J. Kubofcik, T.B. Nutman);; University of Miami Miller School of Medicine, Miami, Florida, USA (J. Cardenas);; Fundación Ecuatoriana Para Investigación en Salud, Quito, Ecuador (P.J. Cooper);; Universidad Internacional del Ecuador, Quito (P.J. Cooper);; St George's University of London, London, UK (P.J. Cooper)

**Keywords:** Ancylostoma ceylanicum, zoonoses, hookworms, soil-transmitted helminths, epidemiology, parasites, zoonoses, Ecuador

## Abstract

*Ancylostoma ceylanicum* hookworms are zoonotic parasites that can infect humans. To detect autochthonous transmission, we analyzed human fecal samples collected in 2000. Multiparallel quantitative PCR detected infection in persons who had never traveled outside Ecuador. These data indicate human transmission of *A. ceylanicum* in the Americas, although endemicity remains unknown.

*Ancylostoma ceylanicum* hookworms can infect humans and have been increasingly recognized as endemic among humans living or traveling to the Asia–Pacific region (*1*). Although *A. ceylanicum* hookworms are known to infect dogs and cats globally, locally acquired human infections have not been reported by persons who have never traveled outside of North or South America. Previous evidence for the potential presence of *A. ceylanicum* hookworms in humans in the Americas is derived from 3 observations: 1) a 1922 autopsy study from northern Brazil identified zoonotic *Ancylostoma* spp. adult hookworms in human intestines when morphologic differentiation of *A. ceylanicum* from *A. braziliense* was still a matter of debate (*2*); 2) a molecular analysis of fecal samples from patients at hospitals in France identified *A. ceylanicum* hookworms in 2 samples, one in a migrant from Colombia and the other in a traveler returning from French Guiana (*3*); and 3) diffuse subacute unilateral neuroretinitis in a child adopted from Colombia and living in Germany was molecularly identified as *A. ceylanicum* infection (*4*). One report describes *A. ceylanicum* identified morphologically in domestic animal populations in South America that originated in Surinam, but no molecular methods were used (*5*).

Such reports, although suggestive, do not provide definitive evidence for autochthonous transmission of *A. ceylanicum* hookworms to humans in the Americas. We used molecular methods to identify autochthonous infections with *A. ceylanicum* in humans who had no history of travel outside Ecuador and who lived in a region of the country where soil-transmitted helminths are endemic. The study protocol was approved by the ethics committees of St George’s Hospital Medical School, London, United Kingdom, and the Foundation Salud y Desarollo Andino, Quito, Ecuador. Informed written consent was obtained from adult participants or from the parents or legal representatives of child participants.

## The Study

In 2000, we collected fecal samples from a convenience subsample of preschool children, schoolchildren, and daycare staff in a larger cross-sectional study of the relationship between soil-transmitted helminth infections and allergy among urban and rural populations living in the Provinces of Pichincha and Esmeraldas, Ecuador. The subsamples represented settings considered to be at high risk for helminth infection (*6*). (Appendix Figure 1). We analyzed single frozen samples from 230 participants with a median age of 9 years (mean age 11, range <1 to 63 years); 40.4% were male and 69.6% were living in an urban setting (town of San Lorenzo). None of the study participants had a history of travel outside Ecuador.

The samples were thawed and ≈250 mg was transferred into collection microtubes in a 96-well format (QIAGEN, https://www.qiagen.com). We performed initial sample lysis by using a TissueLyzer II bead mill (QIAGEN) with 5-mm stainless steel beads. We performed high-throughput DNA extraction on a QiaSymphony (QIAGEN) instrument by using a PowerFecal Pro Kit (QIAGEN), following the manufacturer’s instructions with minor modifications (i.e., samples were pretreated with only the proprietary buffer CD1, and steps involving pretreatment with buffer CD2 were omitted to expedite the high-throughput protocol). All samples were extracted after an internal control was added as reported previously (*7*).

We prepared high-throughput, multiparallel quantitative PCR (qPCR) reactions by using 3.5 μL of Taqman Fast Advanced Master Mix (QIAGEN), 2 μL water, and 1 μL of a PrimeTime qPCR Probe Assay (Integrated DNA Technologies, https://www.idtdna.com) with forward primer, reverse primer, and probe at a final reaction concentrations of 500 nmol/L, 500 nmol/L, and 250 nmol/L, respectively. A 96-well qPCR master plate was prepared for each respective target (*Ascaris lumbricoides/suum* [*8*], *A. ceylanicum* [*9*], *Strongyloides stercoralis* [*10*], *Trichuris trichiura* [*11*], *Necator americanus* [*11*], and *Ancylostoma duodenale* [*11*]), and 6.5 μL of the qPCR solution was transferred to a 384-well plate with a Beckman Coulter liquid handler (Beckman Coulter, https://www.beckmancoulter.com) and subsequently frozen at –80°C until use. After DNA elution, we used the liquid handler to transfer 2 μL of eluted DNA into a 384-well plate preloaded with the qPCR reagents. After brief centrifugation, the sample reactions were run on a ViiaA7 Real Time PCR System (Thermo Fisher Scientific, https://www.thermofisher.com). Genomic DNA or plasmids served as positive reaction controls.

We first amplified the hookworm internal transcribed spacer (ITS) 1, 5.8s, and ITS2 regions by using primers and cycling conditions as described previously (*12*). After PCR amplification, we prepared the amplicon library for sequencing by using the Nanopore Ligation Sequencing Kit (SQK-LSK109; Oxford Nanopore Technologies, https://nanoporetech.com) according to the manufacturer’s instructions. The prepped library was loaded into an R9.4 flow cell and sequenced on a MinION device (Oxford Nanopore Technologies). We base called sequences by using Guppy Software, version 4.5.4 (Oxford Nanopore Technologies), and we determined consensus sequences by using NGSpeciesID with the following parameters: -ont -sample size 300 -m 330 -s 50 -consensus -medaka (*13*). We performed alignment to reference analysis by using Geneious Prime 2021.02 (Biomatters, Ltd, https://www.geneious.com). In addition, the cyclooxygenase 1 region was PCR amplified by using previously reported primers and then Sanger sequenced (Quintara Biosciences, https://quintarabio.com) (*14*). We produced a phylogenetic tree of ITS and *cox1* sequences by using the maximum-likelihood method performed in Geneious Prime 2021.02 with bootstrapping × 1,000 (*15*).

Of samples tested, hookworm infection was detected in 88 (38.3%) samples, of which 85 were infected with *N. americanus* (37.0%), 6 with *A. ceylanicum* (2.6%), and none with *A. duodenale* hookworms. Prevalence rates for other soil-transmitted helminth infections were *Ascaris lumbricoides* (63.9%) *T. trichiura* (69.7%), and *S. stercoralis* (15.2%). Cycle threshold values for *A. ceylanicum*–positive fecal samples detected by qPCR ranged from 25.2 to 37.6. We stratified distributions of sociodemographic factors and soil-transmitted helminth co-infections according to presence versus absence of *A. ceylanicum* or *N. americanus* hookworm infections (Table).

**Table:** Sociodemographic and soil-transmitted helminth co-infection characteristics of 230 persons for whom fecal samples were examined*

Variable	*Ancylostoma ceylanicum*			*Necator americanus*	
	Uninfected, n = 224	Infected, n = 6		Uninfected, n = 145	Infected, n = 85
Median age, y (range)	9 (<1–53)	4 (1–12)		10 (<1–63)	9 (<1–48)
Sex					
M, n = 93	89 (39.7)	4 (66.7)		54 (37.2)	46 (54.1)
F, n = 137	135 (60.3)	2 (33.3)		91 (62.8)	39 (45.9)
Residence					
Rural, n = 70	70 (31.2)	0		25 (17.2)	45 (52.9)
Urban, n = 160	154 (68.8)	6 (100)		120 (82.8)	40 (47.1)
STH co-infections					
*Ascaris lumbricoides*, n = 147	142 (63.4)	5 (83.3)		74 (51.0)	73 (85.8)
*Trichuris trichiura*, n = 160	154 (68.8)	6 (100)		94 (64.8)	66 (77.7)
*Strongyloides stercoralis*, n = 35	32 (14.3)	50.0 (3)		29 (20.0)	6 (7.1)
*N. americanus*, n = 85	82 (36.8)	50.0 (3)		NA	NA
*A. ceylanicum*, n = 6	NA	NA		3 (2.1)	6 (7.1)
No. co-infecting species					
0, n = 22	22 (9.8)	0		22 (15.2)	0
1, n = 62	62 (27.7)	0		59 (40.7)	3 (3.5)
>2, n = 146	140 (62.5)	6 (100)		64 (44.1)	82 (96.5)
*Values are no. (%) except as indicated. Data are stratified according to presence versus absence of *A. ceylanicum* or *N. americanus* hookworm infections. STH, soil-transmitted infection; NA, not applicable.					

We successfully performed hookworm ITS1, 5.8S, ITS2 PCR amplification and amplicon sequencing on 2 of 6 *A. ceylanicum*–positive samples. The consensus sequences (GenBank accession nos. ON773142, ON773142) obtained from these 2 samples were >99% identical to the corresponding unique *A. ceylanicum* sequence targeted by the qPCR (Appendix Figure 2). We prepared a phylogenetic tree comparing known ITS1 sequences of *A. ceylanicum*, *A. duodenale*, *Ancylostoma braziliense*, and *Ancylostoma caninum* hookworms, including the consensus sequences from these 2 positive samples (referred to as being from subjects A and B; Appendix Figure 3). We also compared the phylogeny of *cox1* gene sequences for *A. ceylanicum* hookworms from this study (*A. ceylanicum* accession nos. ON773158) with isolates of the parasite from Asia (Appendix Figure 4).

## Conclusions

We identified *A. ceylanicum* hookworm infection in 6 study participants who lived in an urban setting in Esmeraldas Province and had not traveled outside of Ecuador. All infections were detected in children in a marginalized urban setting where overcrowding is typical and hygiene standards are poor. Although the study was limited by small sample size and geographic restriction, the data provide evidence for autochthonous transmission of *A. ceylanicum* hookworm to humans in the Americas.

Appendix Supplemental results for study of zoonotic *Ancylostoma ceylanicum* hookworm infections, Ecuador.
