# Supplementary material for: Zoonotic Ancylostoma ceylanicum Hookworm Infections, Ecuador
Source: Emerg Infect Dis. 2022 Sep;28(9):1867–9. doi: 10.3201/eid2809.220248 (PMC9423896; doi:10.3201/eid2809.220248)
Supplement: Appendix — Supplemental results for study of zoonotic Ancylostoma ceylanicum hookworm infections, Ecuador. [file 22-0248-Techapp-s1.pdf]

Article DOI: https://doi.org/10.3201/eid2809.220248

Zoonotic *Ancylostoma ceylanicum* Hookworm Infections, Ecuador

Appendix

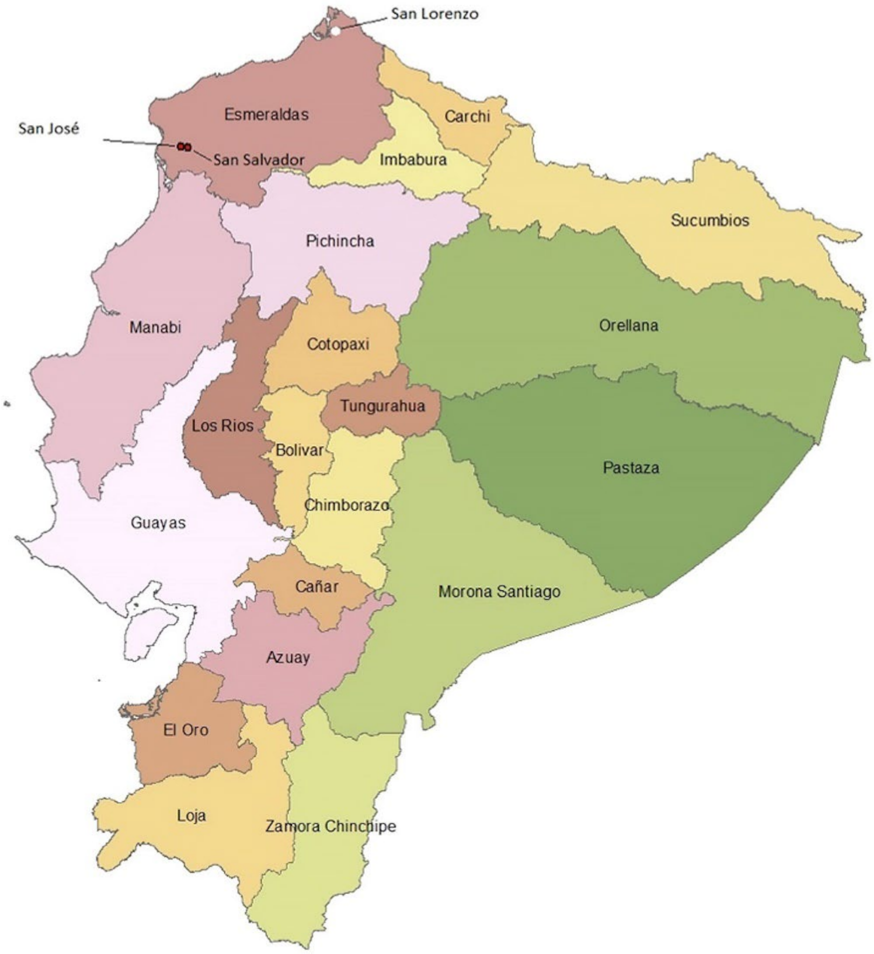

Appendix Figure 1. Locations of samples obtained from Ecuador, indicated by red and white dots.

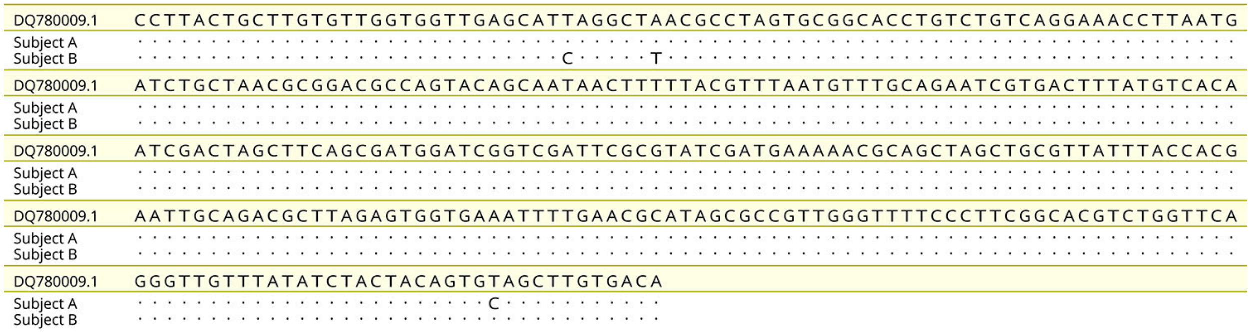

Appendix Figure 2. The alignment of the *Ancylostoma ceylanicum* ITS reference sequence DQ780009.1 and the consensus sequence of the RTHW1 PCR amplicon. The sequences in the top, middle, and lower lane correspond to the amplified region of the reference (Accession #: DQ7800091), patient 5 sample (Acc #: ON773142), and patient 6 sample (Acc#: ON773143), respectively. Nucleotides discordant to the reference are indicated by their corresponding letter. Dots represent agreement with the reference.

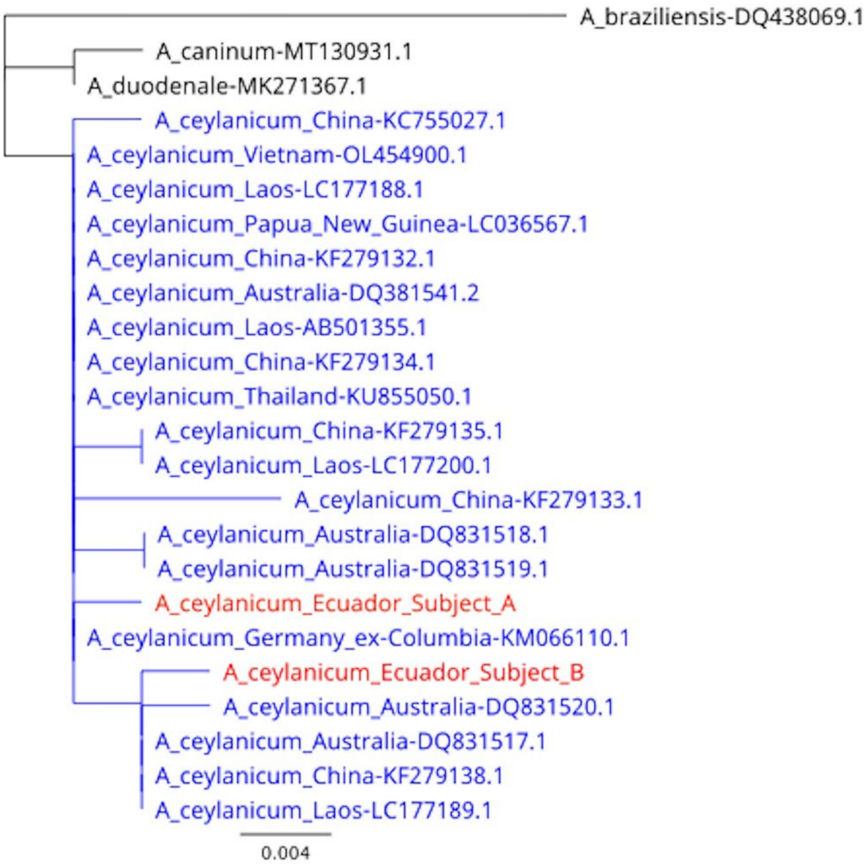

Appendix Figure 3. A phylogenetic tree comparing sequences from *Ancylostoma ceylanicum* infections detected in subjects A and B samples with published ITS1 sequences of *A. ceylanicum*, *A. caninum*, *A. braziliensis*, and *A. duodenale*. The samples in blue are those of *A. ceylanicum* with origin. Sequences from subject samples A and B are indicated in red. Ex-Colombia refers to a sequence obtained from a Colombian migrant living in France.

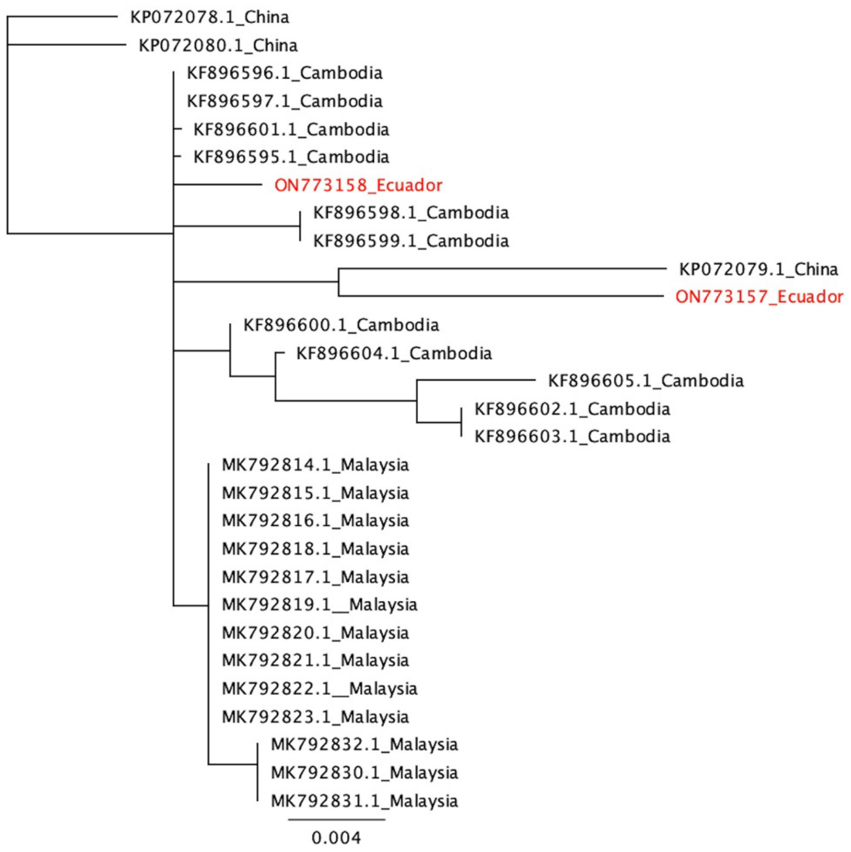

Appendix Figure 4. A phylogenetic tree comparing Cox1 sequences of *Ancylostoma ceylanicum* from subjects A and B with various regions of Asia. The sequences from this study are in red.
